# Event and Comment

**Published:** 1916-01

**Authors:** 


### DENTAL REGISTER

Vol. LXX JANUARY, 1916 No. 1

### EVENT AND COMMENT

OHIO STATE MEETING. The Fiftieth Annual meeting of this Society was held in Columbus, December seventh to tenth, and was in some respects the best meeting in its history. It was not the aim of its officers to celebrate the semi-centennial occasion so much as to have a meeting with the main idea foremost of presenting a program to its members that was up to date in every way. The papers and discussions were high grade and on almost every important topic that is now before the profession. There were a number of short papers on practical subjects that were especially interesting and a series of descriptive clinics that were very instructive. The more than sixty general clinics were perhaps the most interesting feature of the meeting as they enlist the co-operation of a larger number of members of the society and they exemplify individual methods of a more varied character. In our judgment such papers and clinics are of the greatest value because they enlist the interest of the members of the society and thereby increase the internal resources of the society. Then, too, the papers are short and practical in character and appeal to more members than the longer and more formal papers. In fact we think it would be much better if more local and less foreign talent was solicited by our program committees than now seems to be the fad in most of our State societies. The arrangements for holding the sessions in a separate building from the commercial displays was a distinct advantage. The speakers could be heard without difficulty and there was less confusion. The exhibits were given more commodious space than last year and they showed up to much better advantage and attracted much attention. So long as these exhibits are made a part of the program it certainly is better that they be given space for systematic arrangement and display of the supplies and equipment. Many of the displays had demonstrators giving instruction with new appliances and materials which makes the exhibits more valuable and in a sense justifies their place as a part of the program of exercises. The social functions of the society are becoming important features as additional attractions. Theater parties, dinner parties, fraternity banquets, and the general banquet, which is open to every one for the price per plate, keep one busy most of the time, leaving no dull periods; in fact some members are complaining that these social affairs consume too much time. It would seem, however, that a liberal use of the time could be very well given over to these social affairs, as they add interest, and many times things of great value are brought out in these gatherings. It was really a great meeting and the officers are to be congratulated and thanked also for the hard work and wise planning which provided so great a feast of good things of every kind to interest, amuse and instruct. We are sure that no one could attend such a meeting conscientiously and with an open mind but would return to his work a better dentist and an inspiration to make better use of his time in following his calling, and he should also have a deeper consciousness of his duty to his patients.

MILLER MEMORIAL. The unveiling and dedication of the beautiful Miller monument was the most impressive event in connection with the Ohio State meeting. The monument is a life size bronze casting made by Frederick

C. Hibbard, of Chicago. The base is of gray granite on which bronze plates are attached with dates of Dr. Miller's birth and death and the important features commemorated. The monument is placed in front of the Library on the campus of Ohio State University in a beautiful and conspicuous position. A picture of the monument will appear elsewhere in this issue. The dedication address was made by Dr. E. C. Kirk, of Philadelphia, a class mate of Dr. Miller in the old Pennsylvania Dental College. Dr. Truman W. Brophy, President of the International Dental Federation, Dr. Nelville S. Hoff, Dean of the Dental Department of the University of Michigan, Professor Knight, of Ohio University, Dr. Thomas P. Hinman, President of the National Dental Society, and Dr. E. C. Mills, President of the Ohio State Dental Society. Dr. Kirk reviewed the scientific work done by Dr. Miller; Dr. Brophy reviewed the professional work of Dr. Miller in Europe, especially his career as an organizer of co-operative work among the European countries; Dr. Hoff spoke of the coming of Dr. Miller to this country and his plans for completing his life work in his own country; Professor Knight spoke of Dr. Miller's personal record as a fellow class mate and accepted for Ohio State University the memorial; Dr. Hinman spoke of the American appreciation of Dr. Miller's work for the profession of the world and his untimely loss to the American profession. Dr. Mills gave a statement of the history of the monument. Every State Society in the country contributed to the expense, and the monument stands as a memorial of the appreciation by the entire profession of the splendid self-sacrificing work of a noble man. It is probably the finest tribute ever made to a member of the dental profession. The American Dental Society of Europe and Dr. N. S. Jenkins, a great admirer and close friend, sent beautiful floral offerings. Owing to the indefatigable work of Dr. Mills and his committee the monument is all paid for, and will stand for ages as a worthy memorial and an inspiration to students of the science of dentistry.

NATIONAL RESEARCH LABORATORY. Announcement was made at the Ohio meeting by Dr. Weston A. Price, that the trustees of the National Research Commission had purchased a beautiful and substantial building in the city of Cleveland as a permanent home for the research commission. The building is a large and substantial brick and stone house in the finest residence section of Cleveland containing about twenty-five rooms. It is proposed to move into it at once and start researches along several new lines of work. The building cost about fifty thousand dollars and something like sixteen thousand dollars have already been pledged. The Ohio State Society contributed four thousand dollars from its accumulated surplus. This seems like a rather daring move, as it will cost considerable to pay the costs of equipping and maintaining such a plant, but the energy and confidence of Dr. Price and his associates will undoubtedly make a successful venture of it. Is it not possible that some of our wealthy and benevolently inclined laymen could have their attention directed to this most excellent undertaking by the dentists of the country. Here is an excellent chance for some one to continue the excellent examples of the Forsythe Brothers, and Mr. Eastman, of Rochester.

WHO SHOULD TEACH ORAL HYGIENE. A rather spirited discussion has appeared in *Oral Hygiene* as to the propriety of medical men writing articles on mouth sanitation. One writer severely condemning a medical writer for contributing to a daily paper articles on oral sanitation, particularly the mouth toilet, etc. The claim made is that no medical writer would be likely to possess sufficient technical knowledge to present such a subject properly to the public, and also that professional etiquette ought to prevent a medical author from infringing on the provinces of an allied profession. The opposing argument shows that the particular articles were wholly correct and couched in terms that would make a strong appeal to the general newspaper reader. As the writer is well and favorably known, therefore no indictment should be brought against him for using his strong influence in behalf of suffering humanity. Personally we have little sympathy with the constricted views which prohibit any comment either of a personal or public character so long as it is the truth and there is a demand for it which is not adequately supplied through the regular channels. St. Paul said "He would be all things to all men if thereby he could save some." Now in this time of medical criticism of dental practice we are apt to resent excessive interference on the part of our medical confreres, but should we not rather be glad to secure the competent advice and help of our educated sister profession in matters that evidently are to a considerable extent correlated, or which at least have a considerable influence on each other. It will do the medical profession a lot of good to study this problem so that its writings and advice may be competent to reach and influence the general public. We may object to the lay preacher or the uneducated tyro setting forth in the secular prints inaccurate or ill-considered instructions, but there can be no objection to the educated physician interesting himself in methods of oral hygiene.

OFFICERS AND PLACE OF MEETING. Dr. T. I. Way, of Cincinnati, was elected President of the Ohio State Dental Society; Dr. F. M. Casto, of Cleveland, First Vice-President; Dr. C. N. Wright, of Dayton, Second Vice-President; and Drs. J. R. Callahan, of Cincinnati, L. L. Barber, of Toledo, and H. C. Brown, of Columbus, as members of the Board of Directors. The next annual meeting will be held in Toledo. Dayton wanted the next meeting but Toledo seemed to be the better place for the meeting on account of better accommodations and it seemed due to that section of the State.

IMPORTING PROGRAMME TALENT. The following extract for an editorial in the December *New Jersey Dental Journal* is timely and to the point. There are other State societies that need this sort of advice. Will they act upon it? Perhaps, when the boosting idea subsides, and thoughtful men are put on the programme committees. An occasional non-resident with something to say is good for every organization. Excessive help pauperizes.

"We of New Jersey have the reputation of conducting every year one of the greatest and most successful dental conventions held anywhere. Our local societies are all of them up-and-doing organizations, but we have contracted a bad habit, I almost said disease, of importing, as it were, all, or nearly all, of our talent, and shoving our just-as-good home men aside. I ask you now, is this fair? Is it just? Most certainly not. How often do you hear of a Jerseyman reading a paper outside of New Jersey, or, for that matter, in New Jersey? Why isn't he asked to do it? Just because he is not encouraged or given the opportunity to do so before the societies of his own State. Overdrawn? Not a bit. Fourteen local societies hold about ninety meetings during the year, and out of this number perhaps six Jerseymen have been the essayists, the other eighty-four being 'foreigners.'"

